# Lack of PD-L1 Expression by iNKT Cells Improves the Course of Influenza A Infection

**DOI:** 10.1371/journal.pone.0059599

**Published:** 2013-03-15

**Authors:** Hadi Maazi, Abinav K. Singh, Anneliese O. Speak, Vincent Lombardi, Jonathan Lam, Bryant Khoo, Kyung Soo Inn, Arlene H. Sharpe, Jae U. Jung, Omid Akbari

**Affiliations:** 1 Department of Molecular Microbiology and Immunology, Keck School of Medicine, University of Southern California, Los Angeles, California, United States of America; 2 Department of Pathology, Harvard Medical School, Boston, Massachusetts, United States of America; Albany Medical College, United States of America

## Abstract

There is evidence indicating that invariant Natural Killer T (iNKT) cells play an important role in defense against influenza A virus (IAV). However, the effect of inhibitory receptor, programmed death-1 (PD-1), and its ligands, programmed death ligand (PD-L) 1 and 2 on iNKT cells in protection against IAV remains to be elucidated. Here we investigated the effects of these co-stimulatory molecules on iNKT cells in the response to influenza. We discovered that compare to the wild type, PD-L1 deficient mice show reduced sensitivity to IAV infection as evident by reduced weight loss, decreased pulmonary inflammation and cellular infiltration. In contrast, PD-L2 deficient mice showed augmented weight loss, pulmonary inflammation and cellular infiltration compare to the wild type mice after influenza infection. Adoptive transfer of iNKT cells from wild type, PD-L1 or PD-L2 deficient mice into iNKT cell deficient mice recapitulated these findings. Interestingly, in our transfer system PD-L1^−/−^-derived iNKT cells produced high levels of interferon-gamma whereas PD-L2^−/−^-derived iNKT cells produced high amounts of interleukin-4 and 13 suggesting a role for these cytokines in sensitivity to influenza. We identified that PD-L1 negatively regulates the frequency of iNKT cell subsets in the lungs of IAV infected mice. Altogether, these results demonstrate that lack of PD-L1 expression by iNKT cells reduces the sensitivity to IAV and that the presence of PD-L2 is important for dampening the deleterious inflammatory responses after IAV infection. Our findings potentially have clinical implications for developing new therapies for influenza.

## Introduction

Influenza A virus (IAV) infections represent a major public health threat, particularly in the case of children, the elderly and those with underlying diseases, all of whom are at an increased risk for disease complications and death following IAV infection [1], [2]. Seasonal outbreaks alone cause an estimated 200,000 hospitalizations and over 30,000 deaths annually in the United States [3].

Immune system plays an important role in the resolution of IAV infection. Both mucosal and systemic immunity play important roles in the elimination of infection with IAV [4], [5], [6]. Accumulating evidence in the last few years suggests an important role for conventional CD4^+^ and CD8^+^ T cells in the control and clearance of the IAV [7], [8], [9]. However, in recent years, a relatively new T cell population, invariant natural killer T (iNKT) cells, have been reported to act not only as innate lymphocytes but also as regulators of adaptive immune responses [10], [11].

iNKT cells have been suggested to play critical roles in a wide range of immune responses by acting in a pro-inflammatory or anti-inflammatory manner [12], [13]. They are a specialized subset of T lymphocytes expressing markers of the NK cell lineage and an invariant T cell receptor (TCR) [14]. In contrast to conventional T cells, iNKT cells recognize self and exogenous lipid antigens presented by the MHC class I-like molecule CD1d [15], [16]. Upon lipid recognition through their TCR, iNKT cells secrete a range of cytokines with opposing effects on immune responses, which contribute to the activation of NK, T and B cells, and dendritic cells (DCs) [17]. This functional property establishes iNKT cells as innate immune effector cells as well as regulators of adaptive immune responses. Numerous studies have shown that, upon activation, iNKT cells either suppress or enhance immune-mediated responses during inflammation, cancer, autoimmune diseases and infection [15], [18], [19], [20]. There is evidence indicating that iNKT cell responses to viral infection require interaction of iNKT cells with DCs where co-stimulatory interactions may play an important role in determining the outcome of the response.

The PD-1: PD-1 ligand co-stimulatory interaction is a recently characterized signaling pathways within the B7: CD28 superfamily. This co-stimulation consists of the PD-1 receptor and its two ligands PD-L1 (B7-H1) and PD-L2 (B7-DC). PD-L1 is expressed in a wide variety of tissues and by a number of different cell types including T cells, NK T cells and DCs [21], [22], [23], [24], and its expression is up-regulated by IFN- γ [25], [26]. The expression of PD-L2 is much more restricted and appears to be limited to a subset of bone marrow-derived cells, including DCs and macrophages [23], [27]. PD-1 is an inhibitory co-receptor that is expressed on T, iNKT and B cells after activation that delivers an inhibitory signal upon recognition of either of its ligands. Cytokines such as IFN-γ and IL-4 that are produced after T cell activation increase the expression of PD-1 ligands at mucosal surfaces, leading to attenuate the immune response [28]. Although PD-1 has been well characterized as a negative regulator of conventional CD4^+^ T cells, the role of PD-1 and its interaction with PD1 ligands in regulating activation and function of iNKT cells after infection with IAV has not been investigated.

In the present study, we examined the relative contribution of PD-L1 and PD-L2 to the modulation of immune responses after IAV infection. We determined that in the absence of PD-L2 animals succumb more rapidly compared to wild type animals after infection with a lethal dose of IAV virus. This is associated with an increased cellular infiltration into the lungs and enhanced pulmonary inflammation. In contrast, in PD-L1 deficient mice the severity of cellular infiltration and pulmonary inflammation is reduced. This differential regulation of the response to IAV infection is associated with alterations to cytokine production by iNKT cells. These results suggest that under normal circumstances PD-1/PD-L1 interactions are negative regulators of viral clearance, whereas PD-1/PD-L2 interactions are important for dampening deleterious inflammation. Therefore, our findings may have important clinical implications in the sense that targeting PD-1 co-stimulation in vivo may provide a novel therapeutic target for controlling and enhancing host immune responses to IAV allowing an enhanced response with reduced inflammation.

## Materials and Methods

### Mice

Female BALB/c ByJ mice were purchased from Jackson Laboratories. *Jα18* ^−/−^ mice (backcrossed to BALB/c) were a gift from M. Taniguchi/T. Nakayama (Chiba University, Chiba, Japan) and S. Balk (Brigham and Women's Hospital, Boston, Massachusetts) [29]. PD-L1^−/−^, PD-L2^−/−^ and PD-L1^−/−^ PD-L2^−/−^ double knockout mice backcrossed to BALB/cByJ mice for 11 generations were obtained from Arlene Sharpe (Harvard Medical School, Boston, Massachusetts) [30]. Mice were maintained and used according to institutional and National Institutes of Health guidelines in a pathogen-free facility. The Animal Care and Use Committee, University of Southern California approved all animal protocols. A loss of 25% or more of body weight was the criterion for euthanasia according to our IACUC protocol.

### Influenza A infection

Six to eight week-old adult mice were anesthetized with ketamine (200 mg/kg)/xylazine (20 mg/kg) and inoculated intranasally (i.n.) with IAV (strain PR8, H1N1) in 50 μl saline. The virus was grown and harvested from 10-day embryonated chicken eggs as previously described [31]. A dose of 3000 PFU/mouse was chosen as the lethal dose. Control (mock-infected) mice were treated with i.n. allantoic fluid (AF) diluted 1∶500 in saline.

### Flow cytometry analysis

In all experiments, lungs were aseptically removed, minced using sterile razor blades, and incubated in 1.6 mg/ml collagenase (CLS4, Worthington Biochemicals, Lakewood, NJ) and 30 µg/ml DNAse (Sigma-Aldrich, St. Louis, MI) at 37°C for 90 min. To achieve a single-cell suspension, lung fragments were pressed through a 70-µm pore nylon cell strainer using the flat end of a sterile 3-ml syringe plunger. Enzymatic action was terminated by washing cells twice in complete RPMI (RPMI 1640 with L-glutamine and 10% fetal bovine serum (FBS)) by centrifugation at 400× g for 5 min at 4°C. Leukocytes were isolated by centrifugation over a 30–70% Percoll gradient (GE Healthcare, Piscataway, NJ). Cells were then pre-incubated for 30 min. with normal rat serum, and washed before staining. iNKT cells were identified using various antibody combinations that included PE conjugated CD1d: PBS-57 loaded tetramer (NIH, NIAID tetramer core facility, Atlanta GA), TCRβ-allophycocyanin (APC) (clone H57-597, eBioscience, San Diego, CA) and CD4-APC-eFluor750 (clone RM4-5) (eBioscience). Cells were acquired on the FACSCanto II 8 color flow cytometer (BD Biosciences, San Jose, CA) and 10,000 events within the iNKT cell gate were collected. The data were analysed with FlowJo 8.6 software (Tree Star Inc., Ashland, OR).

### Purification of iNKT cells and their sub-populations

Single cell suspensions were prepared from spleen in accordance with standard protocols. Splenocytes were labeled with PE-conjugated CD1d: PBS-57 loaded tetramer for 20 min at 4°C. Cells were then washed and magnetically labeled with anti-PE microbeads (Miltenyi Biotec, Auburn, CA). After washing iNKT cells were obtained by positive selection using an AutoMACS Pro (Miltenyi Biotec) using the Posseld program. This program is designed by the manufacturer for enrichment of rare cells using two cell enrichment columns. Purity of iNKT cells was >80% as determined by flow cytometry analysis. For detection of CD4^+^ and DN iNKT cells in lungs CD3-FITC and CD4-APC-eFluor750 (both eBioscience) were used. For *in vitro* studies, iNKT cells were negatively selected from splenocytes using a cocktail of PE conjugated mAbs against B220, CD62L, CD8α and CD11c, (BD Pharmingen, San Jose, CA) followed by incubation with anti-PE microbeads. The enriched iNKT cell splenocytes were confirmed to contain an average of >10% iNKT cells by tetramer staining and flow cytometry analysis.

### Cytokine production by iNKT cells

Invariant NKT cells were positively selected from lungs of wild type, PD-L1^−/−^ or PD-L2^−/−^ mice five days post infection as described above. iNKT subsets were positively selected and subsequently stained with anti-CD4 antibody and sorted into CD4^+^ and DN subsets by FACS on a BD FACSAria III. Once positively selected iNKT cells were cultured for 48 h and the cells harvested for cytokine determination by quantitative reverse transcription polymerase chain reaction (RT-PCR) as previously described [32]. Briefly, total RNA was extracted from sorted subtypes or total iNKT cells using the RNAeasy mini kit (Qiagen, Valencia, CA) and cDNAs were generated with the High Capacity cDNA Reverse Transcription Kit (Applied Biosystems, Carlsbad, CA) according to the manufacturer's recommendations. Quantification of mRNA levels was carried out by quantitative real-time PCR on a CFX96 thermal cycler (Bio-Rad, Hercules, CA) with predesigned Taqman gene expression assays and reagents, as per manufacturer's instructions.

### Activation of iNKT cells by dendritic cells in vitro

Negatively enriched iNKT cells were cultured in round bottom 96 well plates with 1×10^3^ DCs positively isolated after 3 days from lungs of IAV infected wild type, PD-L1^−/−^ or PD-L2^−/−^ mice, as previously described [13]. CD11c^+^ DCs were isolated using CD11c microbeads (Miltenyi Biotec) according to the manufacturer's instructions. Supernatants were collected after 48 hours and cytokines levels were determined by ELISA (eBioscience).

### Adoptive transfers

Purified iNKT cells (1×10^6^) from PD-L1^−/−^, PD-L2^−/−^ or wild type mice were adoptively transferred into Jα18^−/−^ mice intravenously into the tail vein. Mice were challenged intranasally 24 hours after adoptive transfer with lethal viral dose of 3000 PFU in 50 µl saline.

### BAL fluid analysis

After 5 days of IAV infection, the lungs from PD-L1^−/−^, PD-L2^−/−^ or wild type mice were lavaged twice with 1 ml of PBS with 2% FBS and the fluid pooled as described previously [12]. The relative number of different types of leukocytes was determined from slide preparations of bronchoalveolar lavage (BAL) fluid stained with Hematoxylin and Eosin (H&E).

### Lung Histology

Following 5 days post IAV infection, lungs from the sacrificed mice were perfused with 10% formalin-PBS and removed and fixed immediately in 10% neutral-buffered formalin (v/v). After overnight fixation, the lungs were processed for histology. The lung tissue was embedded in paraffin, 3 µm sections were cut and stained with H&E according to standard protocols. Sections were scanned using light microscope for inflammation. Images of these fields were captured by a Nikon eclipse TE 2000-S microscope (Nikon, USA) objective lens, total magnification 40X, with an in-line camera and assembled into multipanel figures using Adobe Photoshop software (version 7.0). Images were analyzed using the Leica Application suite (Leica Microsystems, Bannockburn, Ill).

### Statistical Analysis

Data were analysed with a one-way ANOVA followed by a Tukey post-test between groups using PRISM v4 software (Graph Pad). P values of less than 0.05 were considered statistically significant with * P<0.05, ** P<0.01 and *** P<0.001. Data represents mean ± SEM.

## Results

### iNKT cells play an important role in the control of influenza viral infection

**Figure 1:**
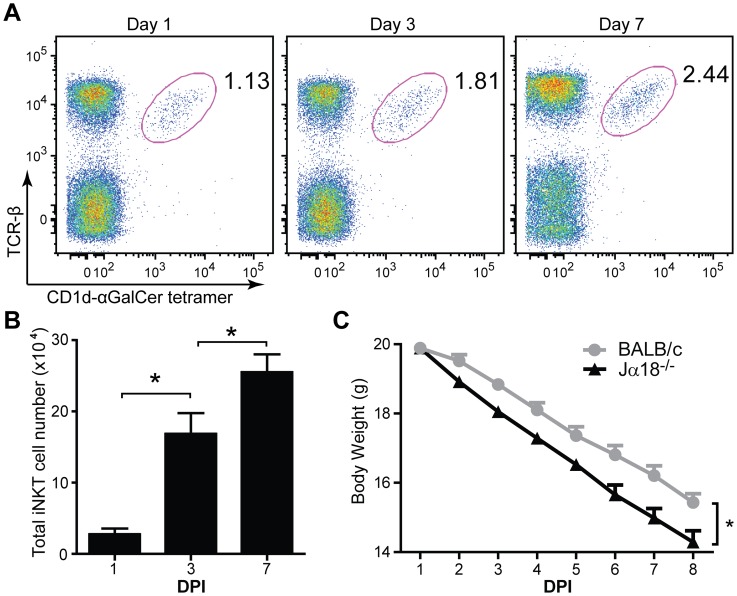
iNKT cells reduce the sensitivity of BALB/c mice to IAV infection. Jα18^−/−^ mice and BALB/c mice (n = 10) were intranasally infected with a lethal dose (3000 PFU) of IAV. (**A**) Increased frequency of iNKT cells after IAV infection. Representative FACS plots of iNKT cell frequencies in the lung of BALB/c mice stained with CD1d: PBS57 loaded tetramer and TCR-β antibody, gated on viable lymphocytes with percentage of cells in the iNKT cell gate indicated next to the gate. (**B**) Total iNKT cell numbers in the lung were determined by flow cytometry at different days post infection (DPI). Data is presented as mean ± standard error of the mean (SEM), n = 3. * P>0.05 (**C**) The weight loss of Jα18^−/−^ mice and BALB/c mice has been monitored daily after IAV infection. Jα18^−/−^ mice show augmented weight loss compared to wild type mice. Data are representative of three independent experiments.

Previous studies have suggested an important role for iNKT cells in the protection against IAV in the C57Bl6/J background [10], [11]. Here we first tested whether iNKT cells are recruited to the lungs after IAV infection and whether these cells play a protective role in mice with BALB/c background. To this end, we first evaluated the frequency and the absolute number of iNK T cells in the lungs of BALB/c mice at day 1, 3 and 7 after infection. As shown in figure 1A and 1B, at day 3 after IAV infection there is a significantly higher number of iNKT cells in the lungs of infected mice compare to day 1, suggesting that IAV infection leads to recruitment of these cells to the lungs. At day 7 after infection, there is even higher frequency and number of iNKT cells in the lung compare to day 1 and 3.

We next addressed the role of iNKT cells in reducing the sensitivity to IAV by infecting wild type BALB/c and Jα18^−/−^ mice that lack iNKT cells and evaluated the loss of body weight as an index for influenza progression. As shown in figure 1C, we found that although initially both strains had the same body weight, after the second day post infection Jα18^−/−^ mice display severer weight loss compare to wild type BALB/c mice (P<0.05). These data suggest that iNKT cells play a role in reducing the sensitivity to IAV infection also in BALB/c background and that this role of iNKT cells is not strain dependent.

### PD-1 and PD-L1 expression on iNKT cells are modulated in response to IAV infection

**Figure 2:**
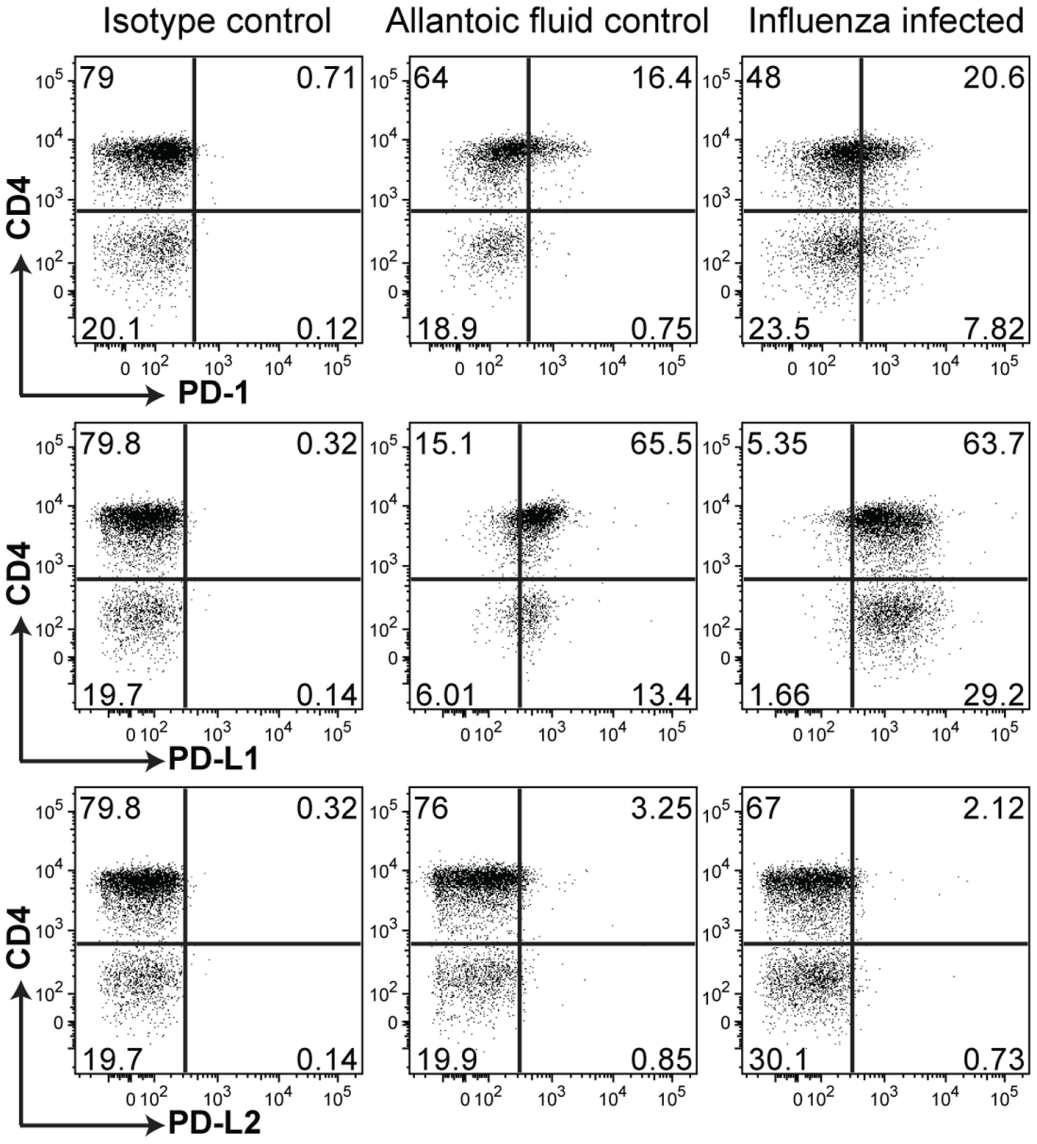
Differential expansion of iNKT cell subsets to IAV infection. Expression of PD-1, PD-L1 and PD-L2 on iNKT cells isolated from the lungs of control or 5 day post IAV infection in BALB/c mice. Dot plots are gated on iNKT cells that were identified as CD1d: PBS57 loaded tetramer^+^ TCR-β^intermediate^ and show CD4 expression on the iNKT cells (Dot plots are representative of 5).

After confirming the role of iNKT cells in reducing the sensitivity to IAV, we addressed the role of co-stimulatory molecule PD-1 and its ligands on iNKTcells. It is known that iNKT cells can express PD-1 and PD-L1 but do not express PD-L2. [13]. We sought to determine whether the expression of these molecules is modulated after IAV infection. To reach this aim, the expression of PD-1, PD-L1 and PD-L2 by iNKT cells subsets was assessed by flow cytometry 5 days after IAV infection. As shown in figure 2, expression of PD-1 is increased in CD4^+^ iNKT cells in IAV infected mice compare to mock treated control mice. Interestingly, double negative iNKT cells of IAV infected mice show even a greater expression of PD-1 compare to mock treated mice. Similarly, the level of PD-L1 expression by CD4^+^ and by double negative iNKT cells is higher in IAV infected compare to control mice. As expected, neither CD4^+^ nor double negative iNKT cells express PD-L2 in IAV infected or control mice. These data suggest that the protective role of iNKT cells may be mediated by co-stimulatory molecule PD-1 and its ligands.

### PD-L1 and PD-L2 modulate sensitivity to IAV infection in opposing directions

**Figure 3:**
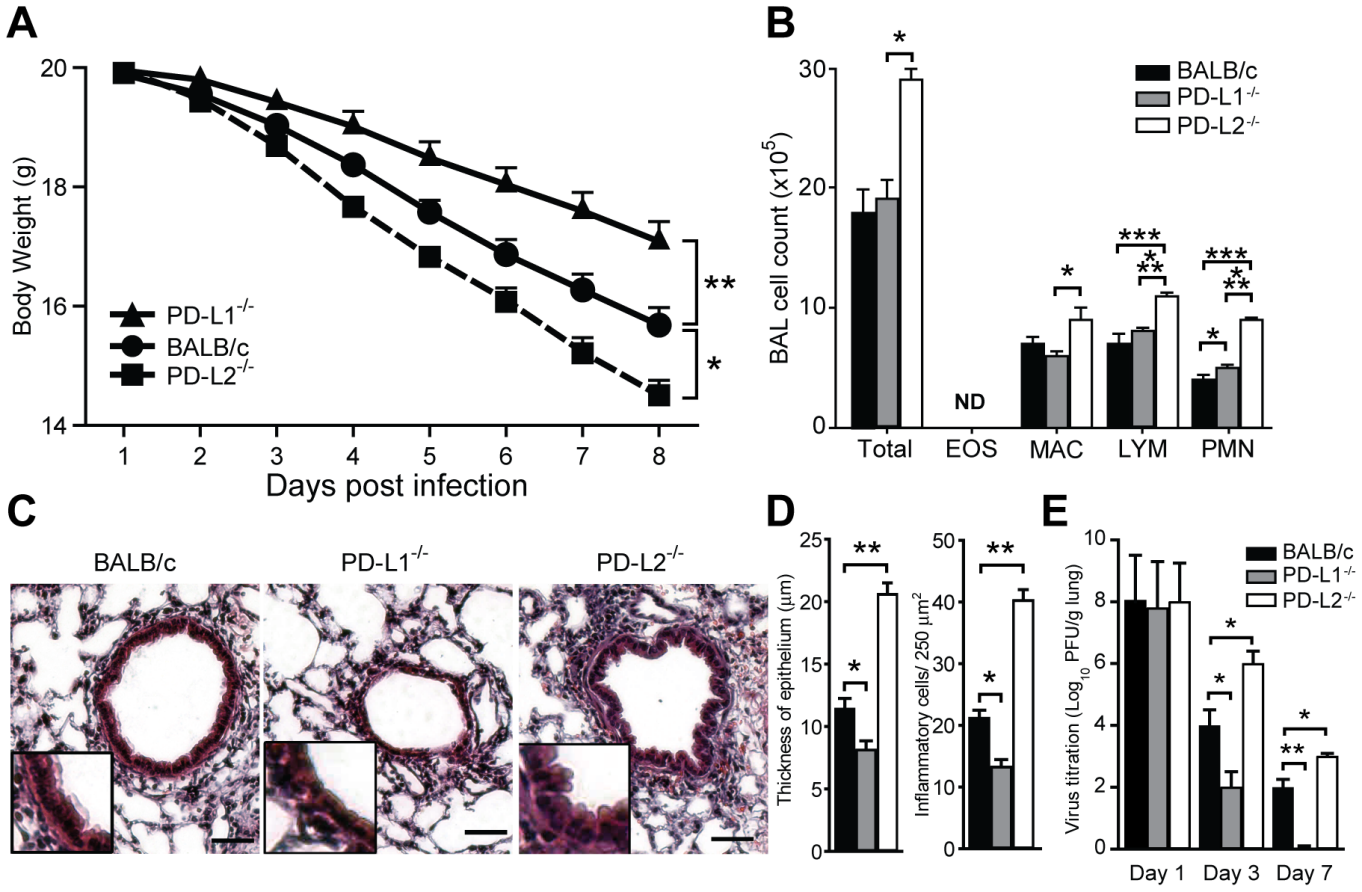
PD-L1 and PD-L2 affect the sensitivity and the degree of lung inflammation after infection with IAV. PD-L2^−/−^, PD-L1^−/−^ and BALB/c (n = 10) mice received a lethal dose of IAV (3000 PFU) intranasally. (**A**) Weight loss has been monitored by measuring body weight daily post infection (DPI) (**B**) BAL was performed in mice (n = 5) five days post IAV infection and analyzed for differential cellular infiltration. Bar graph shows total cell number (Total) and absolute number of eosinophils (EOS), monocytes/macrophages (MAC), lymphocytes (LYM) and neutrophils (PMN). ND: not detectable. (**C**) Representative image of lung sections taken 5 days post infection with 3000 PFU IAV stained with H&E (original magnification ×40, insert magnification ×100, scale bar 50 μm) from BALB/c, PD-L1^−/−^ and PD-L2^−/−^ mice. (**D**) Quantification of lung sections as shown by measurement of thickness of epithelium, left panel and number of inflammatory cells per 250 µm^2^, right panel. (**E**) Virus titration in the lungs shown as plaque forming units (PFU) per gram of lung tissue. Data are presented as mean ± SEM, * P>0.05, ** P>0.01 and *** P>0.001 as calculated by a one-way ANOVA with Tukey post hoc test and is representative of four independent experiments.

Next we examined the contribution of PD-1 ligands to the sensitivity to IAV infection at the functional level. To this end, we compared the severity of IAV infection in wild type BALB/c with mice lacking PD-L1 and those lacking PD-L2. Body weight loss, the composition of BAL, lung histology and virus titer in the lungs were assessed as indications of influenza severity. We found that in the absence of PD-L1, mice show significantly higher body weight as compared to wild type mice (P<0.05, Fig. 3A). In contrast, mice lacking PD-L2 show a greater weigh loss than wild type mice (P<0.05, Fig. 3A) indicating that PD-L1 contributes to sensitivity to IAV infection.

The increased weight loss of PD-L2^−/−^ mice was associated with a significant increase in the number of macrophages and lymphocytes in BAL compared to wild type mice (P<0.05 and P<0.001, Fig. 3B). Histological analysis of lungs revealed that compare to wild type mice there is an increased number of inflammatory cells and epithelium thickness in the lungs of PD-L2^−/−^ mice whereas, there is a decreased number of inflammatory cells and epithelium thickness in the lungs of PD-L1^−/−^ mice (P<0.05, Fig. 3C–D). Similarly, the lungs of PD-L1^−/−^ mice contain a significantly lower titer of IAV at day 3 and 7 post infection compare to wild type mice. IAV titer in lungs of PD-L2^−/−^ mice is significantly higher compare to wild type mice at 3 and 7 days post infection.

Since we observed that PD-L1 and PD-L2 have opposing effects, we addressed the response to IAV infection in the absence of both of these co-stimulatory molecules. To this end, we infected PD-L1^−/−^ PD-L2^−/−^ double knockout or wild type BALB/c. Interestingly, we found that the there is no difference in the level of weight loss between wild type and PD-L1^−/−^ PD-L2^−/−^ double knockout mice (figure S1). These data suggest that PD-L1 and PD-L2 have the capacity to balance/neutralize each others effect and therefore, the absence of both results in no phenotype in IAV resistance or sensitivity.

Taken together, these results indicate that PD-L1^−/−^ contributes to the sensitivity to infection and its severity, while PD-L2^−/−^ plays a role in recovery and protection against IAV.

### Lack of PD-L1 expression on iNKT cells reduces sensitivity to IAV infection

**Figure 4:**
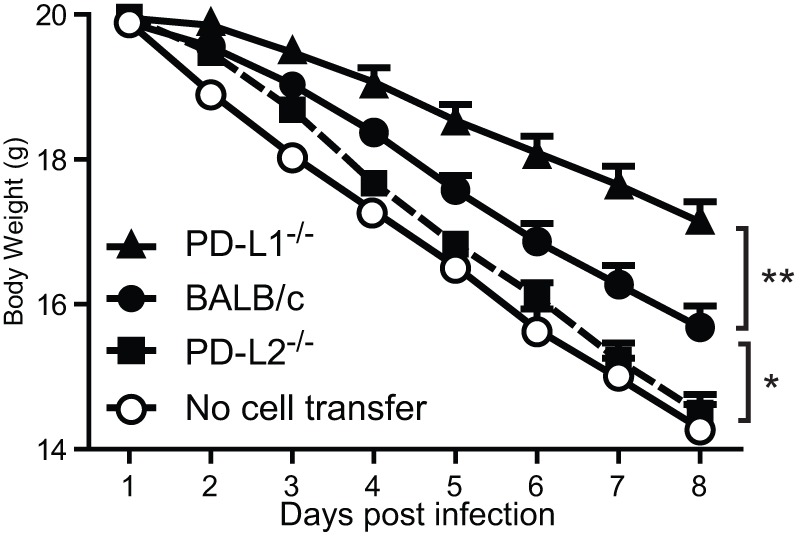
Lack of PD-L1 expression on iNKT cells reduces sensitivity to IAV infection. iNKT cells (1×10^6^) were positively isolated from spleen of BALB/c, PD-L1^−/−^ and PD-L2^−/−^ mice and adoptively transferred into iNKT cell deficient Jα18^−/−^ mice. After 24 hours Jα18^−/−^ mice received 3000 PFU influenza A intranasally. Weight loss was monitored at different time points after the infection with n = 5 per group and is representative of three independent experiments.

To determine whether the expression of PD-1 ligands on iNKT cells or development of these cells in the absence of PD-1 ligands underlies the altered sensitivity to IAV we performed a series of adoptive transfer experiments. iNKT cells were positively selected from the spleen of BALB/c, PD-L1^−/−^ or PD-L2^−/−^ animals and adoptively transferred into iNKT cell deficient Jα18^−/−^ mice. As expected Jα18^−/−^ mice that did not receive any iNKT cells had a high rate of weight loss that was reduced after the transfer of wild type iNKT cells (P<0.05, Fig. 4), confirming the essential role of these cells. In contrast mice that received PD-L2^−/−^ iNKT cells demonstrated no significant difference in weight loss compare to animals that did not receive iNKT cells (Fig. 4). Whereas, transfer of PD-L1^−/−^ iNKT cells resulted in enhanced protection from IAV as evident by the reduced weight loss compared to animals that received BALB/c iNKT cells and PD-L2^−/−^ iNKT cells (P = 0.01, Fig. 4). These data clearly demonstrate that expression of PD-L1 specifically by iNKT cells contributes to influenza pathogenesis and severity. Moreover, our results suggest that although iNKT cells do not express PD-L2, their protective role against IAV requires the presence of this co-stimulatory molecule during the development of iNKT cells.

### Cytokine profile of pulmonary iNKT cells is regulated by PD-L1 and PD-L2 co-stimulation

**Figure 5:**
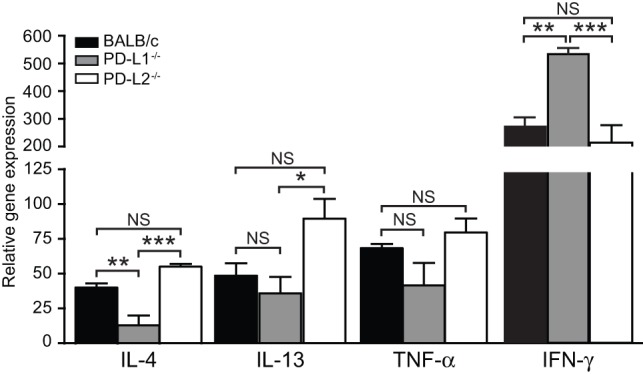
PD-L1 and PD-L2 affect the cytokines produced by lung iNKT cells after IAV infection. iNKT cells were positively selected from influenza A infected lungs on day 5 from PD-L1^−/−^, PD-L2^−/−^ and wild type BALB/c mice and different cytokines were analysed by quantitative RT-PCR normalized to β-actin levels. Data presented as mean ± SEM. * P>0.05, ** P>0.01 and *** P>0.001 as calculated by a one-way ANOVA with Tukey post hoc test and is representative of five independent experiments with n of 5 per group.

Our adoptive transfer studies suggest that even though iNKT cells do not express PD-L2 they are functionally affected by development in a PD-L2 deficient environment. It has been previously reported that iNKT cells from PD-L1 or PD-L2 knockout mice have altered cytokine polarization [13]. Therefore, we sought to determine how PD-L1 and PD-L2 signaling might affect the function of iNKT cells in response to IAV infection. Lung iNKT cells from IAV infected PD-L1^−/−^ mice produced significantly higher levels of IFN-γ compared to wild type BALB/c and PD-L2^−/−^ mice (Fig. 5). In contrast, higher levels of IL-4 were produced from IAV infected PD-L2^−/−^ mice lung iNKT cells compared to PD-L1^−/−^ lung iNKT cells (Fig. 5). PD-L1^−/−^ lung iNKT cells also expressed significantly less IL-4 compared to wild type BALB/c mice after IAV infection (Fig. 5). These experiments indicate that signaling through PD-L1 and PD-L2 can skew the cytokines produced by pulmonary iNKT cells in response to IAV infection either towards more antiviral (PD-L1) or less antiviral (PD-L2) profile. The inflammatory cytokine, IL-13, was also significantly higher in PD-L2^−/−^ mice compared PD-L1^−/−^ mice (Fig. 5) whereas there was no significant difference in TNF-α between the groups of mice (Fig. 5).

### PD-L1 and PD-L2 expression on dendritic cells regulates the iNKT cell after influenza infection

**Figure 6:**
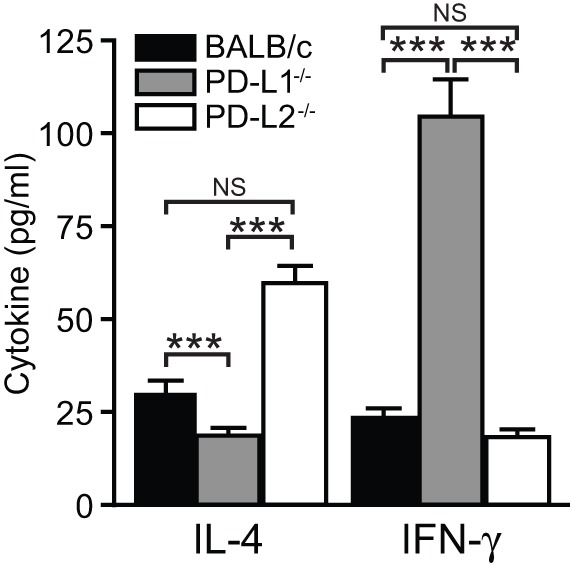
PD-L1 and PD-L2 expression on dendritic cells regulates the iNKT cell cytokine secretions after IAV infection. Pulmonary DCs (10^3^) were isolated from lungs of influenza A infected PD-L1^−/−^, PD-L2^−/−^ and wild type BALB/c mice and loaded with 1 µg of α-galactosylceramide for 3 hours and then cultured with negatively enriched iNKT cells (1×10^4^) from the spleen of naïve wild type mice. Supernatants were collected after 48 h and IL-4 and IFN- γ levels were measured by ELISA. Data presented as mean ± SEM. *** P>0.001 as calculated by a one-way ANOVA with Tukey post hoc test and is representative of four independent experiments with n of 5 per group.

As demonstrated above, cytokine production by iNKT cells is altered by the absence of PD-L1 or PD-L2. As both PD-L1 and PD-L2 can be expressed by DCs [13], [33], [34] we sought to determine the role of DC expression of these co-stimulatory molecules on cytokine secretion by iNKT cells. DCs were isolated from the lungs of IAV infected PD-L1^−/−^, PD-L2^−/−^ and BALB/c mice and loaded with an iNKT cell stimulating ligand, α-galactosylceramide, prior to co-culture with BALB/c iNKT cells. When using PD-L1^−/−^ DCs production of IFN-γ was increased compared to BALB/c or PD-L2^−/−^ DCs (Fig. 6). In contrast, PD-L2^−/−^ DCs resulted in enhanced production of IL-4 compared to wild type and PD-L1^−/−^ mice (Fig. 6). Therefore, PD-L1 and PD-L2 can exert effects on the cytokine profile of iNKT cells through their expression on the DCs that present antigens to and activate iNKT cells.

### Lack of PD-L1 leads to preferential expansion of double negative iNKT cell in IAV infected mice

**Figure 7:**
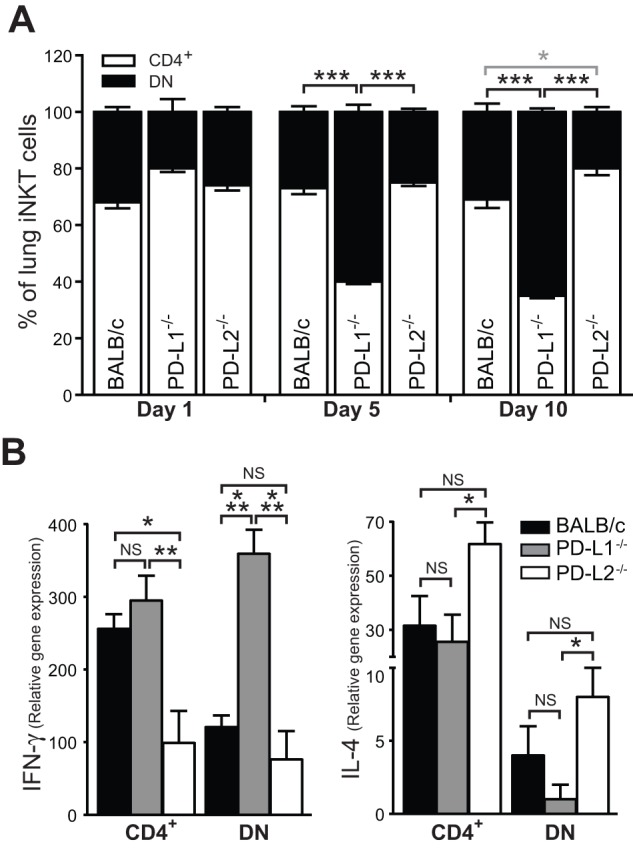
iNKT cell subpopulations are modulated in PD-L1^−/−^ and PD-L2^−/−^ animals after IAV infection. (**a**) Wild type BALB/c, PD-L1^−/−^ and PD-L2^−/−^ mice (n = 3) were infected with IAV intranasally. Lungs were collected at day 1, 5 and 10 post infection and the frequency of CD4^+^ and DN iNKT cells determined by flow cytometry and compared (DN: black asterisk, CD4^+^: gray asterisk). (**b**) CD4^+^ and DN iNKT cells subsets were sorted from IAV infected lungs on day 5 from PD-L1^−/−^, PD-L2^−/−^ and wild type mice BALB/c and IL-4 and IFN- γ were analysed by quantitative RT-PCR normalized to β-actin levels (n = 8). Data presented as mean ± SEM. * P>0.05, ** P>0.01 and *** P>0.001 as calculated by a one-way ANOVA with Tukey post hoc test and is representative of five independent experiments.

Murine iNKT cells may be either CD4^+^CD8^−^ (CD4^+^) or CD4^−^CD8^−^ (DN) and it has been previously demonstrated that these different iNKT cell populations produce a different profile of cytokines [35], [36]. Therefore, we reasoned that the differences in the cytokines produced by the pulmonary iNKT cells in PD-L1^−/−^ and PD-L2^−/−^ compared to wild type could be due to differential composition of iNKT cell subpopulations. Hence, we determined the relative proportions of CD4^+^ and DN iNKT cells in the lung of IAV infected BALB/c, PD-L1^−/−^ and PD-L2^−/−^ at various days post infection. At five days post infection the frequency of DN iNKT was increased in the PD-L1^−/−^ animals compared to BALB/c and PD-L2^−/−^ mice (Fig. 7A). By day 10, the expansion of DN iNKT cells in the lung of PD-L1^−/−^ was even greater and significantly different compared to wild type BALB/c and PD-L2^−/−^ mice (Fig. 7A). Furthermore, in the lung of PD-L2^−/−^ mice the frequency of CD4^+^ iNKT cells was significantly increased compared to wild type BALB/c mice (gray asterisk, Fig. 7A). These finding are in agreement with a recent study that demonstrated the protective role of DN iNKT cells during the resolution of IAV infection [35]. We reasoned that the protective role of DN iNKT cells was associated with enhanced IFN-γ secretion. Therefore, we analyzed IFN-γ gene expression of iNKT cell subsets following IAV infection. As shown in figure 7B both iNKT cell subpopulations from PD-L1^−/−^ mice demonstrated enhanced IFN- γ expression compared to PD-L2^−/−^ mice. The expression of IFN-γ by the DN iNKT cell subset from PD-L1^−/−^ mice was significantly higher than wild type BALB/c mice (Fig. 7B) with BALB/c CD4^+^ iNKT cells expressing significantly more IFN-γ compared to CD4^+^ iNKT cells from PD-L2^−/−^ mice (Fig. 7B). In contrast, IL-4 was increased in both iNKT cell subsets from PD-L2^−/−^ compared to PD-L1^−/−^ mice (Fig. 7B). These findings indicate that the role of iNKT cells in reducing the severity of IAV in absence of PD-L1 co-stimulation is associated with the increased DN iNKT cell frequency and enhanced IFN-γ production whereas in the absence of PD-L2 CD4^+^ iNKT cells are increased resulting in the production of more IL-4.

## Discussion

Our findings suggest an important role for the PD-1 ligands, PD-L1 and PD-L2 in regulating the immune response to infection with IAV. In the absence of PD-L1 mice are less sensitive to IAV infection as evident by reduced excessive cellular infiltration and inflammation and weight loss. In contrast, in the absence of PD-L2 pulmonary inflammation and cellular infiltration is increased and results in a more rapid weight loss. This altered response is associated with the production of different cytokines by iNKT cells. Adoptive transfer of iNKT cells from PD-L1 or PD-L2 deficient mice to animals lacking iNKT cells recapitulated the findings from the whole animal. Interestingly, we found that PD-L1 and PD-L2 have possibly balancing/neutralizing effects on each other as PD-L1^−/−^ PD-L2^−/−^ double knockout mice show a similar sensitivity to IAV infection as do wild type mice. We also identified a role for PD-1/PD-L interactions in controlling the expansion of iNKT cell subsets. In the absence of PD-L1 DN iNKT cells were increased whereas the opposite occurred in mice lacking PD-L2 with an increase in CD4^+^ iNKT cells. We have also demonstrated an important role for expression of PD-1 ligands on DCs in the control of cytokine production by iNKT cells. Altogether these results indicate that PD-1/PD-L1 interactions are vital modulators of the immune response and represent novel targets for therapeutic modification in vivo.

The role of iNKT cells in the control of viral infections is well characterized. In terms of viral immunity, iNKT cells are involved in immune surveillance and/or clearance of hepatitis B and C virus [37], [38], respiratory syncytial virus [39], herpes simplex virus 1 and 2 [40], [41], and HIV [42]. Several reports have indicated that iNKT cells are important in IAV clearance. In both murine infection models and human patients it has been demonstrated that iNKT cells are required for the generation of adaptive immune responses by reducing the suppressive effects of myeloid derived suppressor cells, which expand after infection [10]. Furthermore, activation of iNKT cells with a potent agonist, α-galactosylceramide, improves the disease course in part through enhancing innate immune responses [43]. iNKT cells have also been demonstrated to play an important role in the generation of influenza specific CD8^+^ T cell responses [11]. With all these findings it is not surprising that iNKT cell deficient Jα18^−/−^ mice are more sensitive to IAV, exhibit a higher rate of weight loss and increased severity of disease. This influence of iNKT cells on multiple aspects during the course of IAV infection and clearance are likely associated with the ability of iNKT cells to produce both Th1 and Th2 cytokines very rapidly after activation [44]. Mounting evidence now suggests that two subsets of iNKT cells, based on their expression of CD4 or not (CD4^+^ or DN), are enriched in the production of certain cytokines [36], [45]. Therefore, it is interesting that after infection with IAV the relative proportions of iNKT cell subsets in the lungs are altered in the absence of PD-L1 or PD-L2.

In PD-L1^−/−^ mice the frequency of DN iNKT cells, that are the major producers of IFN-γ, is increased. In contrast, in PD-L2^−/−^ mice the proportion of CD4^+^ iNKT cells is increased resulting in the enhanced production of IL-4. Thus, not only does signaling through the PD-1/PD-L pathway influence the cytokines production by the iNKT cells but also controls the proportions of the iNKT cell subsets. The underlying mechanism for this finding is not clear as yet but may have important implications in attempts to polarize immune responses in a Th1 or Th2 biased manner. In addition to iNKT cells, previous reports suggest that T cells, in particular CD8^+^ T cells, play a major role in producing IFN-γ that assists in the clearance of IAV [46], [47]. However, recent studies provide evidence that iNKT cells are important in the generation of influenza specific CD8^+^ T cells responses [11]. Here, we found that iNKT cells from PD-L1^−/−^ mice showed increased IFN-γ production after IAV infection. There is evidence indicating that IFN-γ possesses different opposing functions including pro inflammatory Th1 responses and immuneregulatory function depending on the cytokine milieu and the state of immune responses [48], [49], [50]. We also found that pulmonary iNKT cells from PD-L2^−/−^ mice produce more IL-4 and IL-13 after influenza infection. Although not directly tested at the functional level, our findings suggest that the altered cytokine profile of iNKT cells in the absence of PD-L1 or PD-L2 may underlie the observed difference in IAV sensitivity. During pulmonary inflammation Th2 cells produce IL-4 and IL-13 [24], which interact with mast cells or eosinophils to mediate the inflammatory responses. Earlier, Moran and coworkers reported that treatment of mice with IL-4 resulted in a significant delay in viral clearance [51]. A recent report suggested that pulmonary IL-13 is responsible for the viral induced lung inflammation and airway hyperreactivity [52]. Acute viral infection leads to the production of IL-13 which has been shown to be responsible for many characteristics of asthma [52]. In our experiments we show that lack of PD-L2 is associated with increased production of IL-13 by iNKT cells which contributes to the augmented pathogenesis of IAV infection in these mice. However, on the other side, it is well established that Type I interferons are key cytokines produced by IAV-infected epithelial cells and monocytes and play an important role in clearance of virus [53], [54], [55].

The role of the molecules PD-L1 and PD-L2 in the activation and modulation of iNKT cells in influenza pathogenesis has not previously been investigated. The PD-1: PD-L pathway is best known for its ability to negatively regulate immune responses [33], [56]. Most of the evidence for this role comes from models of tolerance, cancer or chronic infections [30], [57], [58], [59], [60]. In the present study, we observed that PD-L1 expression modulates the cytokine profile of iNKT cells and consequently regulates the inflammatory phase after influenza infection. Nevertheless, our findings regarding the role of PD-1 ligands is focused on the acute phase of the influenza and the impact of these ligands on the pathogenesis of influenza during the late phase remains to be elucidated.

The PD-1 ligands exhibit distinct patterns of expression. Previous studies have shown that PD-L1 is expressed more broadly than PD-L2. PD-L1 is expressed on various hematopoietic and non-hematopoietic cells [21], [22], [33], whereas, expression of PD-L2 is predominantly on dendritic cells and macrophages [61]. It has been reported that PD-1 and PD-L1 are expressed in the lower airways of patients with IAV infection [62]. Interestingly, we found that IAV infection significantly, increases the expression of PD-1 and PD-L1 by DN respiratory iNKT cells. However PD-L2 expression by iNKT cells was minimal.

Our in vitro experiments would suggest that even though PD-L2 is not expressed on iNKT cells, development of iNKT cells in a PD-L2 deficient environment primes them towards exhibiting a Th2 like cytokine profile. This is demonstrated in the effects of adoptively transferred PD-L2^−/−^-derived iNKT cells on the severity of IAV infection in iNKT cell deficient mice. To further characterize what cell type could express PD-L2 and may be responsible for altering the cytokine production by iNKT cells we studied DCs. In a co-culture model naïve wild type iNKT cells activated by PD-L2^−/−^ DCs demonstrated the same bias of cytokine production towards IL-4 and IL-13. Taken together, our findings suggest that development of iNKT cells in the absence of PD-L2 affects their phenotype towards reduced antiviral characteristics, whereas, lack of PD-L1 expression by iNKT cells or development of these cells in the absence of PD-L1 primes them towards an enhanced antiviral phenotype.

Resolution of influenza virus requires a potent anti-viral immune response on the one hand and regulating the amplitude of the elicited immune response on the other hand. The control of the balance between viral clearance and inflammation is a delicate process that is not fully understood to date. Here we have confirmed the important role of iNKT cells in the host immune response after influenza infection and have extended these findings to the influence of the PD-1/PD-L pathway on the iNKT cell response. Our data indicates that lack of PD-L1 improves the course of influenza disease, whereas the presence of PD-L2 provides signals to control the deleterious inflammation. Therefore, treatment strategies that target these pathways, including block PD-L1 or enhancing PD-L2 signaling, represent novel methods for modulating the host's response after influenza infection that allow more rapid viral clearance with minimized inflammation.

## Supporting Information

Figure S1 PD-L1^−/−^ PDL2^−/−^ double knockout show a similar sensitivity to IAV as wild type BALB/c. (a) PD-L1^−/−^ PDL2^−/−^ double knockout and wild type BALB/c mice were infected with IAV (3000 PFU). Weight loss is monitored daily. Data presented as mean ± SEM.
(EPS)
